# Phytocontact dermatitis due to Ranunculus arvensis mimicking burn injury: report of three cases and literature review

**DOI:** 10.1186/1865-1380-4-7

**Published:** 2011-02-21

**Authors:** Sami Akbulut, Heybet Semur, Ozkan Kose, Ayhan Ozhasenekler, Mustafa Celiktas, Murat Basbug, Yusuf Yagmur

**Affiliations:** 1 Department of Surgery, Diyarbakir Education and Research Hospital, 21400, Diyarbakir, Turkey; 2 Department of Surgery, Ergani State Hospital, Ergani, Diyarbakir, Turkey; 3 Department of Orthopaedics and Traumatology, Diyarbakir Education and Research Hospital, 21400, Diyarbakir, Turkey; 4 4Department of Emergency Medicine, Diyarbakir Education and Research Hospital, 21400, Diyarbakir, Turkey

## Abstract

*Ranunculus arvensis *(corn buttercup) is a plant species of the genus *Ranunculus *that is frequently used in the Far East to treat rheumatic diseases and several dermatological disorders. In Turkey, the plant is seen in the eastern and southeastern Anatolian highlands, which are underdeveloped areas of the country. Herein, we report three patients who used *Ranunculus arvensis *for the treatment of arthralgia and osteoarthritis. A distinctive phytodermatitis developed on the right thumb in one patient (48-year-old male), on the anterior aspect of both knees in another patient (70-year-old female) and all around both knees in a third (59-year-old female). The patients were treated with topical antibiotics and daily wound dressing, and none of them experienced any complications. *Ranunculus arvensis *was confirmed as the cause of the phytodermatitis in the three cases. Poultices of plants applied to the skin demonstrate beneficial effects on many dermatological and rheumatic diseases; however, they have several adverse effects that should not be ignored. In this study, we also present a review of 25 cases reported in the literature.

## Introduction

Burn injuries can be encountered in all ages. The most common burn injuries among the Turkish population are caused by a variety of causes: fires, scalding substances (i.e., traditional Turkish tea, hot milk, etc.), electricity and chemical agents. When taking into account the mechanisms of chemical burns, it was observed that 4% of cases were caused by the application of herbs used as traditional medication [1]. Despite the advances in medicine, a tendency towards using alternative treatments can be seen in every population, including the Turkish one, and plant application is among the most common methods used in folk medicine.

*Ranunculus arvensis *(a member of the *Ranunculaceae *family) is a wild plant traditionally used in the Far East to treat arthritis, asthma, gout, high fever and psoriasis, and is highly allergenic in spring during the flowering period. In Turkey, the plant is frequently seen in the high mountains of the Mediterranean region and the southeastern and eastern regions of Anatolia, which are agricultural areas with plant production [2-11]. Herein, we present three patients with chemical burns caused by *Ranunculus arvensis *used as poultice around the knees and the thumb for the treatment of rheumatic symptoms.

## Case reports

### Case 1

**Figure 1:**
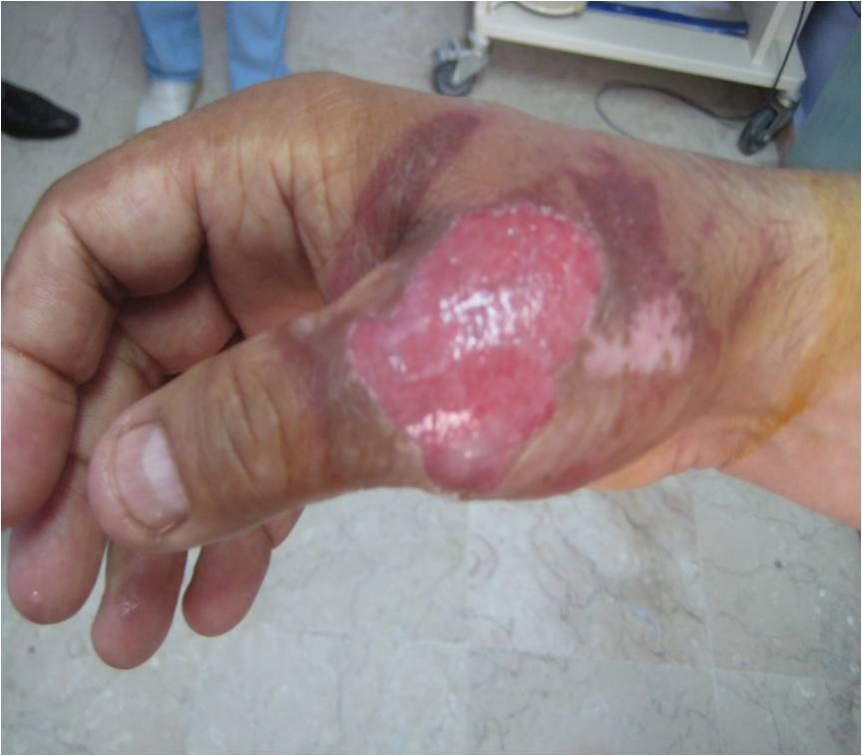
**Phytocontact dermatitis on right thumb (case 1)**.

A 48-year-old man was admitted to our emergency department because of an open wound on his right thumb (Figure 1). Following a neighbor's advice, the patient had applied bruised plant material as a poultice to his right thumb, covering it with an occlusive bandage for 1 h to treat arthralgia. This procedure had resulted in pain and bullous and erythematous lesions on the treated area. The patient did not apply any other substance to the wound and left it open. One day later, as there was no improvement, the patient presented to our clinic and was hospitalized. The lesion healed within 3 weeks with appropriate topical fusidic acid therapy and daily dressing changes. The plant specimens provided by the patient were identified in the Department of Pharmaceutical Botany, Faculty of Pharmacy, at Marmara University as *Ranunculus arvensis*, a member of the *Ranunculaceae *family.

### Case 2

**Figure 2:**
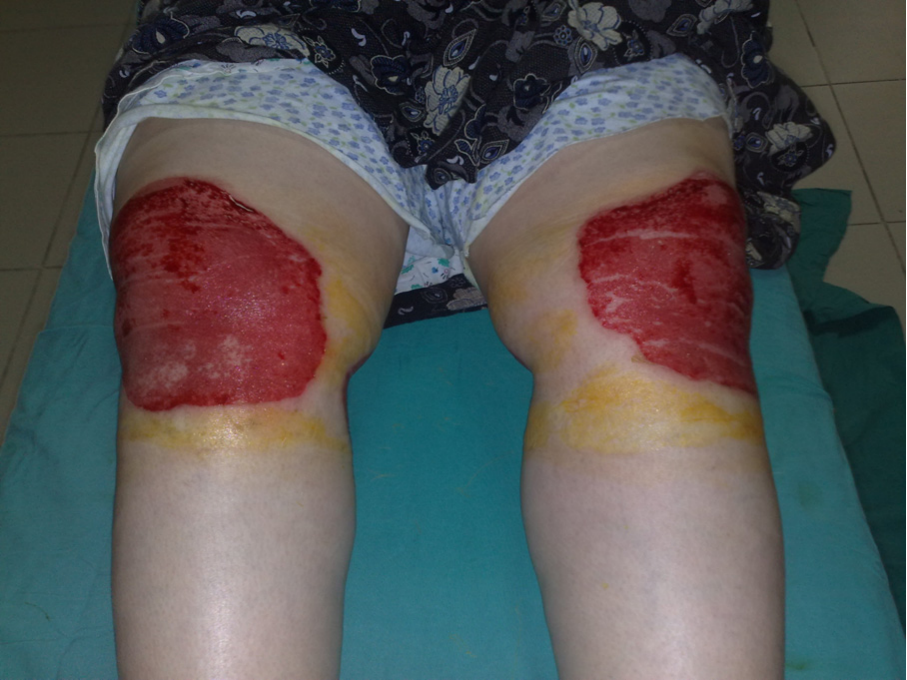
**Phytocontact dermatitis on both knees mimicking burn injury (case 2)**.

A 59-year-old female patient presented to our burn unit with complaints of vesiculo-bullous lesions that were circumferential around both knees (Figure 2). Three days before, at the recommendation of a neighbor, she had applied plant paste on her both knees, covering them overnight for osteoarthritis-related pain. When unfurling the bandages, the patient had noticed wounds over the treated areas. As no improvement had occurred after 3 days, the patient presented to our clinic. Routine laboratory investigations revealed values within normal ranges, and radiological examination showed no pathological findings. On physical examination, all vital signs were stable. Because the patient had diabetes mellitus managed by diet alone, cefazolin sodium was started as antibiotic prophylaxis. The patient was hospitalized in the burn unit, and the wounds were washed with chlorhexidine scrub. When the debris and bullous lesions were removed, second-third degree skin injuries were observed. The lesion healed within 2 weeks with appropriate topical silver sulfadiazine cream and daily dressing changes. No contracture developed during the 4-month follow-up period. The plant specimens provided by the patient were identified as *Ranunculus arvensis*.

### Case 3

**Figure 3:**
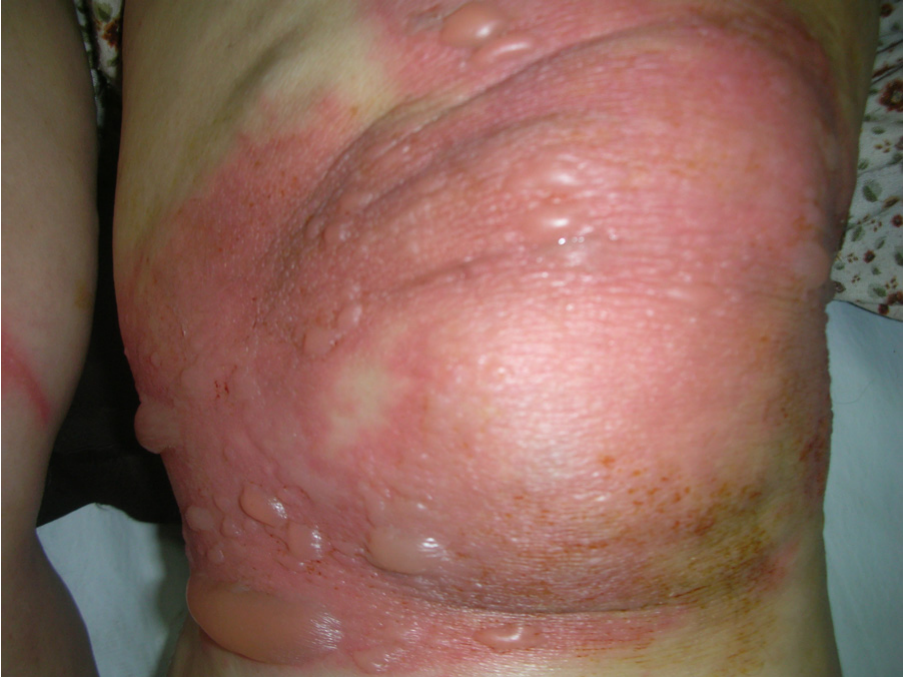
**Phytocontact dermatitis on both knees mimicking burn injury (case 3)**.

A 70-year-old woman living in a rural area of Diyarbakir presented to our emergency outpatient unit with marked burns on both knees (Figure 3). According to the history, the patient, suffering from bilateral knee pain not responding to analgesics, had followed the recommendation of a neighbor; she ground a plant found growing in the mountains and applied it to both knees. Despite the pain, she had not unfurled the bandages for 2 days, and after removing the poultices, she had noticed burn wounds. On the same day, the patient presented to our emergency unit. Her medical history revealed no chronic disease except hypertension. On physical examination, second-degree burns on the anterior aspect of both knees were observed. After performing debridement on the first day of admission, the injuries were cleaned with chlorhexidine scrub and topical silver sulfadiazine cream. By the end of the 10th day, the patient had recovered completely. The plant specimens were identical with those in the first two cases.

## Discussion

The plants of the genus *Ranunculus *contain the toxic glycoside ranunculin. In case of dermal contact, ranunculin is broken down to protoanemonin, which leads to dermal-epidermal separation and formation of bullous lesions. This clinical condition is called phytodermatitis [4,8,10].

Protoanemonin is a volatile and highly vesicant oil, whose toxicity may be explained by the increase in free oxygen radicals resulting in the inhibition of DNA polymerase. The irritant effect of protoanemonin is highest during spring when the plant is blooming and has fresh leaves, and decreases to a minimum as the plant dries up [3]. All three patients reported in this study presented to our clinic in spring.

Members of the *Ranunculaceae *family are widely used as traditional treatment in the form of poultices for various medical conditions, such as abscess drainage, bullous lesions, hemorrhoids, burns and lacerations, and in the form of herbal remedies for rheumatic and myalgic pain, common colds, etc. [2,8-10].

In the literature, the terms "plant burn" and "phytodermatitis" have been frequently used interchangeably. Metin et al. [8] proposed the name 'phytodermatitis' to designate this medical condition; however, in our opinion, the important point is not the name, but how it is treated. After all, the above-mentioned two terms interpret alterations in the anatomic integrity of the skin with pathogenic mechanisms resembling those of burn injury. Therefore, treatment plans should be made in accordance with the methods for treating burns.

Eskitascioglu et al. [4] noted in their study that the severity of chemical burns caused by plant poultices depends on the application method and duration. Reviewing the literature, we found that most patients used the plant as a poultice that was applied to the painful extremity and was covered with a cloth for a period ranging from 25 min to 48 h. We assume that this covering method increases the rate of contact and the degree of damage.

When scanning the literature using PubMed and the Google scholar database, we accessed ten articles on phytocontact dermatitis caused by plants from the *Ranunculaceae *family. A total of 25 patients--18 females and 7 males--aged between 17 and 76 years (mean age: 53.4 ± 14.1 years) were presented in these studies. Twenty-one patients were living in the eastern and southeastern regions, and four in the western regions of Turkey. Age, gender and clinical data for the patients are summarized in Table 1. As shown in the table, women are two times more likely to use alternative medicine than men. Our experience supports this observation, and we postulate that it might be due to the fact that women are more prone to follow the advice of their neighbors and to trust folk medicine.

**Table 1:** Age, gender and clinical characteristics for 25 cases of phytocontact dermatitis caused by plants of the Ranunculaceae family and mimicking burn injuries (25 reported in the literature and our 3 cases)

Ref.	Age	Sex	Implementation period	Admission to hospital	Location	Type of plant	Approach to lesions	Healing time
2	64	M	12 h	Immediately	Left distal thigh	*R. arvensis*	Debridement, topical nitrofurantoin	3 weeks

3	17	M	48 h	2 days	Back, scrotum, penis, chest	*R. arvensis*	Wet dressing, silver sulfadiazine, collogenase	4 weeks

4	42	M	8 h	1 week	Left foot dorsum and ankle	*C. testiculatus*	Clorhexidine scrub + split thickness skin graft	7 days
	
	40	F	4 h	3 weeks	Right foot dorsum and ankle	*C. testiculatus*	Clorhexidine scrub + paraffin gauze	10 days
	
	60	F	2 h	10 days	Right foot dosrum and left knee	*C. testiculatus*	Clorhexidine scrub + paraffin gauze	7 days
	
	65	F	2 h	1 week	Left knee	*C. testiculatus*	Clorhexidine scrub+ paraffin gauze	15 days
	
	48	F	4 h	14 days	Right leg	*C. falcatus*	Clorhexidine scrub + paraffin gauze	2 weeks

5	52-76	F:6 M:3	12 h	NA	Both knees: 7 One knee: 2	*R. constantinopolitanus*	Topical antibacterial treatment	10 d

6	55	F	1 day	2 days	Right knee	R. illyricus	Wet dressing and topical antibiotics	4 days

7	58	F	2 days	5 days	Left knee	*R. illyricus*	Topical antibacterial cream	A few days
	
	54	F	1 days	3 days	Right knee	*R. illyricus*	Wet dressing and topical antibiotic	1 week

8	69	M	2.5 h	2 days	Left knee	*C. falcatus*	Wet dressing and topical fusidic acid	2 weeks
	
	33	F	1.5 h	2 days	Right distal leg, ankle, dorsal foot	C. falcatus	Wet dressing and topical antibiotic	3 weeks
	
	18	F	1 h	1 week	Left ankle, dorsal foot	*C. falcatus*	Wet dressing and topical antibiotic	2 weeks

9	47	F	25 min	NA	Right knee	*C. falcatus*	Wet dressing and topical mometasone cream	10 days

10	45	F	Overnight	2 days	Abdomen, right leg	*R. damascenus*	Wet dressing and topical fusidic acid	10 days

11	NA	F	NA	NA	Right ankle	*C. falcatus*	Wet dressing	2 weeks

**Current**	48	M	1 h	1 days	Right thumb	*R. arvensis*	Dressing with fusidic acid	3 weeks
	
	59	F	Overnight	3 days	Bilateral knee	*R. arvensis*	Clorhexidine scrub + silver sulfadiazine cream	2 weeks
	
	70	F	2 days	Immediately	Bilateral knee	*R. arvensis*	Clorhexidine scrub + silver sulfadiazine cream	10 days

In addition, the results of this literature scan revealed that people living in socio-culturally and economically underdeveloped regions are more enthusiastic about using alternative treatment methods. All of the patients presented in this study were living in a culturally backward area located in a mountainous and rural region of southeastern Turkey. As we have often observed, herbal products are frequently used for the purpose of treating psoriasis, hemorrhoids, back/lower back pain and arthralgia. This may be explained by the fact that folk medicine is an easily accessible, affordable and natural form of treatment; also, there is still a lack of reliance on pharmaceuticals as well as a desire to avoid long waiting times in the hospital.

Burn injuries are still a major cause of mortality and morbidity in most of the developing world, with burn wound infections being the most important complication. Loss of the normal skin barrier, as well as impairment of many systemic host-defense mechanisms, makes burn wounds susceptible to colonization and infection by multiple endogenous microorganisms. The patient remains vulnerable to invasive infection until the wound is completely epithelialized [12]. Therefore, the areas with disrupted skin integrity should be covered as soon as possible, and, for this purpose, grafting and topical antibacterial dressing are most commonly used in the early stages. Reviewing the literature, we observed that in most of the reported cases, antimicrobial dressings were applied, and the predominantly used agents in burn wound care were: silver sulfadiazine, fusidic acid, mafenide, nitrofurazone, chlorhexidine, povidone-iodine, mupirocin, etc. In our burn unit, we frequently prefer dressings containing an antimicrobial agent to cover the burn wound.

In conclusion, although plant poultices applied to the skin show positive effects on many dermatological and rheumatic diseases, they also have many adverse effects. We believe that benefiting from modern medicine is the correct approach rather than attempting alternative treatment methods, whose therapeutic effects have not been proven yet by scientific studies.

## Consent

Written informed consent was obtained from the patients for publication of this case report and accompanying images. A copy of the written consent is available for review by the Editor-in-Chief of this journal

## Competing interests

The authors declare that they have no competing interests.

## Authors' contributions

AS, KO,CM and BM made the daily dressings; AS, YY, OA and SH contributed to writing the article and reviewing the literature as well as undertaking a comprehensive literature search; AS, BM, KO, SH and CM contributed to the design of the study and manuscript preparation.

## References

[B1] Sakallioglu AE Başaran O Tarim A Turk E Kut A Haberal M Burns in Turkish children and adolescents: nine years of experience Burns 2007 33 1 46 51 10.1016/j.burns.2006.05.003 17084031

[B2] Orak M Ustundag M Guloglu C Tas M Baylan B A skin burn associated with Panunculus arvensis Indian J Dermatol 2009 54 19 20 10.4103/0019-5154.45435

[B3] Sayhan MB Gokdemir MT Guloglu C Orak M Ustundag M A Burn case associated with Ranunculus arvensis Anatol J clin Investig 2009 3 1 85 87

[B4] Eskitascioglu T Dogan F Sahin G Ozkose M Coruh A Ozyazgan I An extraordinary chemical burn injury cause: buttercup, a report of five cases Burns 2008 34 5 727 30 10.1016/j.burns.2007.01.016 17624679

[B5] Kose R Okur MI Bingol I Cetin H Phytocontact dermatitis mimicking a burn injury due to Ranunculus constantinopolitanus Contact Dermatitis 2008 59 4 249 50 10.1111/j.1600-0536.2008.01368.x 18844703

[B6] Polat M Oztas P Yalcin B Tamer E Gur G Alli N Contact dermatitis due to Allivum sativum and Ranunculus illyricus: two cases Contact Dermatitis 2007 57 4 279 80 10.1111/j.1600-0536.2007.01120.x 17868226

[B7] Oztas P Gur G Senlik B Yalcin B Polat M Tamer E Alli N Phytocontact dermatitis due to Ranunculus illyricus: two cases J Eur Acad Dermatol Venereol 2006 20 10 1372 3 10.1111/j.1468-3083.2006.01725.x 17062085

[B8] Metin A Calka O Akdeniz N Behçet L Phytodermatitis from Ceratocephalus falcatus Contact Dermatitis 2005 52 6 314 6 10.1111/j.0105-1873.2005.00587.x 15932581

[B9] Karaca S Kulac M Kucuker H Phytodermatitis caused by Ceratocephalus falcatus (Ranunculacea) Eur J Dermatol 2005 15 5 404 5 16172054

[B10] Metin A Calka O Behçet L Yildirim E Phytodermatitis from Ranunculus damascenus Contact Dermatitis 2000 44 3 183 10.1034/j.1600-0536.2001.440308-4.x 11217994

[B11] Yenidünya MO Can Z Demirseren ME A burn from a plant Plast Reconstr Surg 1999 103 1 335 6 9915212 10.1097/00006534-199901000-00076

[B12] Palmieri TL Greenhalgh DG Topical treatment of pediatric patients with burns: a practical guide Am J Clin Dermatol 2002 3 8 529 34 10.2165/00128071-200203080-00003 12358554

